# Patient Adherence to Scheduled Vital Sign Measurements During Home Telemonitoring: Analysis of the Intervention Arm in a Before and After Trial

**DOI:** 10.2196/medinform.9200

**Published:** 2018-04-09

**Authors:** Branko Celler, Ahmadreza Argha, Marlien Varnfield, Rajiv Jayasena

**Affiliations:** ^1^ Biomedical Systems Research Laboratory University of New South Wales Sydney, New South Wales Australia; ^2^ Health and Biosecurity Business Unit eHealth Research Program Commonwealth Scientific and Industrial Research Organisation Parkville, VIC Australia

**Keywords:** patient compliance, vital signs, telehealth, telemonitoring, clinical trial, chronic disease

## Abstract

**Background:**

In a home telemonitoring trial, patient adherence with scheduled vital signs measurements is an important aspect that has not been thoroughly studied and for which data in the literature are limited. Levels of adherence have been reported as varying from approximately 40% to 90%, and in most cases, the adherence rate usually dropped off steadily over time. This drop is more evident in the first few weeks or months after the start. Higher adherence rates have been reported for simple types of monitoring and for shorter periods of intervention. If patients do not follow the intended procedure, poorer results than expected may be achieved. Hence, analyzing factors that can influence patient adherence is of great importance.

**Objective:**

The goal of the research was to present findings on patient adherence with scheduled vital signs measurements in the recently completed Commonwealth Scientific and Industrial Research Organisation (CSIRO) national trial of home telemonitoring of patients (mean age 70.5 years, SD 9.3 years) with chronic conditions (chronic obstructive pulmonary disease, coronary artery disease, hypertensive diseases, congestive heart failure, diabetes, or asthma) carried out at 5 locations along the east coast of Australia. We investigated the ability of chronically ill patients to carry out a daily schedule of vital signs measurements as part of a chronic disease management care plan over periods exceeding 6 months (302 days, SD 135 days) and explored different levels of adherence for different measurements as a function of age, gender, and supervisory models.

**Methods:**

In this study, 113 patients forming the test arm of a Before and After Control Intervention (BACI) home telemonitoring trial were analyzed. Patients were required to monitor on a daily basis a range of vital signs determined by their chronic condition and comorbidities. Vital signs included noninvasive blood pressure, pulse oximetry, spirometry, electrocardiogram (ECG), blood glucose level, body temperature, and body weight. Adherence was calculated as the number of days during which at least 1 measurement was taken over all days where measurements were scheduled. Different levels of adherence for different measurements, as a function of age, gender, and supervisory models, were analyzed using linear regression and analysis of covariance for a period of 1 year after the intervention.

**Results:**

Patients were monitored on average for 302 (SD 135) days, although some continued beyond 12 months. The overall adherence rate for all measurements was 64.1% (range 59.4% to 68.8%). The adherence rates of patients monitored in hospital settings relative to those monitored in community settings were significantly higher for spirometry (69.3%, range 60.4% to 78.2%, versus 41.0%, range 33.1% to 49.0%, *P*<.001), body weight (64.5%, range 55.7% to 73.2%, versus 40.5%, range 32.3% to 48.7%, *P*<.001), and body temperature (66.8%, range 59.7% to 73.9%, versus 55.2%, range 48.4% to 61.9%, *P*=.03). Adherence with blood glucose measurements (58.1%, range 46.7% to 69.5%, versus 50.2%, range 42.8% to 57.6%, *P*=.24) was not significantly different overall. Adherence rates for blood pressure (68.5%, range 62.7% to 74.2%, versus 59.7%, range 52.1% to 67.3%, *P*=.04), ECG (65.6%, range 59.7% to 71.5%, versus 56.5%, range 48.7% to 64.4%, *P*=.047), and pulse oximetry (67.0%, range 61.4% to 72.7%, versus 56.4%, range 48.6% to 64.1%, *P*=.02) were significantly higher in males relative to female subjects. No statistical differences were observed between rates of adherence for the younger patient group (70 years and younger) and older patient group (older than 70 years).

**Conclusions:**

Patients with chronic conditions enrolled in the home telemonitoring trial were able to record their vital signs at home at least once every 2 days over prolonged periods of time. Male patients maintained a higher adherence than female patients over time, and patients supervised by hospital-based care coordinators reported higher levels of adherence with their measurement schedule relative to patients supervised in community settings. This was most noticeable for spirometry.

**Trial Registration:**

Australian New Zealand Clinical Trials Registry ACTRN12613000635763; https://www.anzctr.org.au/Trial/Registration/TrialReview.aspx?id=364030&isReview=true (Archived by WebCite at http://www.webcitation.org/6xPOU3DpR).

## Introduction

Telehealth, the delivery of health services at a distance, has been extensively studied in various at-home, primary care, and hospital-based settings for more than 20 years [1-5]. Large health care organizations such as the Veterans Administration in the United States or the National Health Service in the United Kingdom have already adopted a range of telehealth solutions [6]. The aging population and increasing burden of chronic disease together with the availability of low-cost monitoring technology have resulted in increasing interest in deploying telehealth services for the management of patients with chronic conditions and an increasing market demand worldwide for telehealth services.

Different aspects of telehealth solutions, such as clinical, service, and economic benefits, have been investigated in several pilot projects [7,8]. These factors need to be evaluated in order to promote wide-scale implementation and reduce costs. However, patient adherence with scheduled vital signs measurements or their use of technology is an important aspect that has not been thoroughly studied. As patients in home telehealth programs may choose to stop their participation [9] or may not follow the intended procedure, poorer results than expected may be achieved [10]. In order to gain the maximum benefits of at-home telehealth services, adherence rates should be high. Hence, analyzing factors that can influence patient adherence is of great importance.

Relatively few randomized controlled trials on at-home telemonitoring of vital signs have been reported [3,11]. This paper reports findings on patient adherence in the recently completed Commonwealth Scientific and Industrial Research Organisation (CSIRO) trial of home monitoring for chronic disease management, carried out at several locations along the east coast of Australia [12]. The trial was designed to explore a wide set of outcomes resulting from the introduction of a telehealth model of service based on at-home telemonitoring of vital signs and administration of a range of clinical questionnaires to patients with a range of chronic conditions supervised in either hospital-based or community-based settings.

The clinical protocols for the trial [12], the data architecture design [13], decision support and statistical trend analysis of vital signs data [14], and the impact of telemonitoring on health care expenditure, hospital admissions, and length of stay [8] have been previously published.

In hospital intensive care units, it is well recognized that serious adverse events can be prevented [15-20] by recognizing early warning signs of clinical and physiological deterioration and responding appropriately. The authors concluded that early recognition of these events presents an opportunity for decreasing mortality. There is thus a clear analogy between the use of early warning systems in intensive care units and emergency departments and the early warning that is available through the longitudinal monitoring of vital signs at home of chronically ill individuals.

However, there is currently limited knowledge about the influences and determinants of home telehealth adherence in frail elderly people and their carers [21,22]. Their ability to adhere to a strict regime of telemonitoring has been identified as an important influence on the success of telemonitoring programs [23], and there is a perception that patients will not adhere to a monitoring protocol and will often abandon the program [24,25].

A systematic review of the uptake and continued use of telehealth for patients with congestive heart failure (CHF) and chronic obstructive pulmonary disease (COPD) [26] reported that almost one-third of patients who were offered telehealth services refused and one-fifth of patients who did accept subsequently withdrew. Other studies on the adherence and effectiveness of at-home telemonitoring [27] report that over 1 year the adherence rate for recording weight was 75% and for the recording of blood pressure, 90%, while another study [28] reported values of 12% to 75% for adherence with daily weighing for CHF patients. According to Maeder et al [6], reported levels of adherence varied from approximately 40% to 90%. In most of these studies, the adherence rate usually dropped off steadily over time. This drop is more evident in the first few weeks or months after the start. Higher adherence rates have been reported for simple types of monitoring and for shorter periods of intervention [6]. As an illustration, while adherence rates of 89% were reported by Port et al [29] for the first 2 months, this rate dropped to around 50% within 10 months. The adherence rate in a more complex monitoring system after lung transplantation was about 42% [30].

In this paper we undertake a comprehensive analysis of patient adherence with their measurement schedules in the CSIRO National Telehealth Trial, driven by the hypothesis that longitudinal vital signs data and periodic patient-administered clinical questionnaires provide powerful tools for early identification of an exacerbation of a patient’s condition and permit the early mobilization of clinical resources to avoid unnecessary hospitalization.

## Methods

### Research Ethics Committee Approval

The clinical trial protocol for this study was approved by the CSIRO Human Research Ethics Committee (approval number 13/04, March 25, 2013) as well as 5 other state- and site-based local ethics committees.

### Patient Selection

A master register of 1429 eligible patients was formed from hospital lists provided by local health districts and patients known to clinical staff. Local health districts were located in the states of Queensland (QLD), New South Wales (NSW), the Australian Capital Territory (ACT), Victoria (VIC), and Tasmania (TAS). Subjects were eligible to participate in the study if they met inclusion criteria that were comprehensively described in an earlier publication [11] but mentioned briefly here for convenience: age 50 years and older; 2 or more unplanned acute admissions during the last 12 months or 4 or more unplanned acute admissions during the previous 5 years; and a principal diagnosis of COPD, coronary artery disease, hypertensive diseases, CHF, diabetes, or asthma. From the master register, 479 patients were still deemed eligible after individual screening and were contactable. Patients were deemed ineligible if they had been diagnosed with compromised cognitive function, a neuromuscular disease, cancer, or a psychiatric condition. Note that we chose to take a population health approach to patient selection in that all patients who met the inclusion criteria were eligible, irrespective of their chronic condition. As a result, the patient cohort is subsequently treated as a homogenous group in this paper.

**Figure 1:**
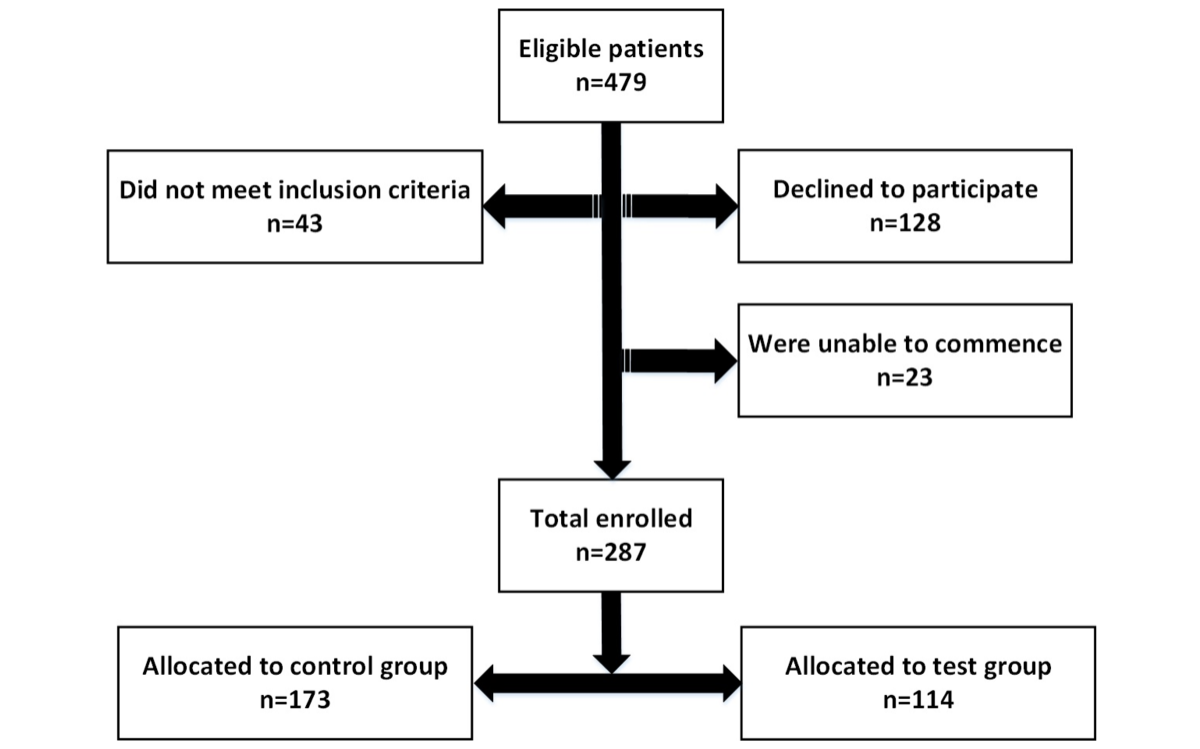
Final cohort of test and control patients.

As reported in Celler et al [8], of the 479 eligible patients, 128 (26.7%) declined to participate, 41 patients did not meet inclusion criteria at interview, and 23 were unable to commence. Of the remaining 287 subjects who consented, 114 were randomly allocated to the telemonitoring test group and the remaining 173 were allocated to the control group (Figure 1). In addition, it was found that vital sign records for 1 subject were missing. Test patients were supplied with a telemonitoring system and trained on its use on installation, while the control group continued to receive normal care through their primary care physician.

### Organization of Care

A project officer at each test site was responsible for managing operational and research activities for the study, thereby separating patient care from study operations. The PO was also responsible for consenting patients, onsite visits, equipment maintenance, and technical support.

The clinical care coordinators (CCCs) were experienced nursing staff, seconded part-time from each trial site health service provider. In 2 of the 5 trial sites, the CCCs were specialist nurses based in local hospitals and able to draw upon the clinical resources of the hospital. The remaining 3 sites were community sites located within primary care organizations called Medicare Locals. In these sites, the CCCs were community nurses with little access to additional clinical resources other than the patients’ own primary care provider.

The CCCs monitored patients’ vital signs and clinical questionnaire responses recorded via the telemonitoring unit daily during business hours. In response to exacerbations in their patients’ conditions, as evidenced by changes in the patients’ vital signs and questionnaire responses, CCCs would initiate and coordinate a timely response to avoid a further exacerbation of their condition and possible hospitalization. Time spent by patients taking their vital signs at home and time spent by CCCs in managing the patients under their care was monitored by comprehensive logs routinely collected by the telemonitoring system and clinician portal. CCCs could also report time spent on a professional portal set up to provide a chat room for CCCs and POs.

### Vital Signs Telemonitoring Unit

**Figure 2:**
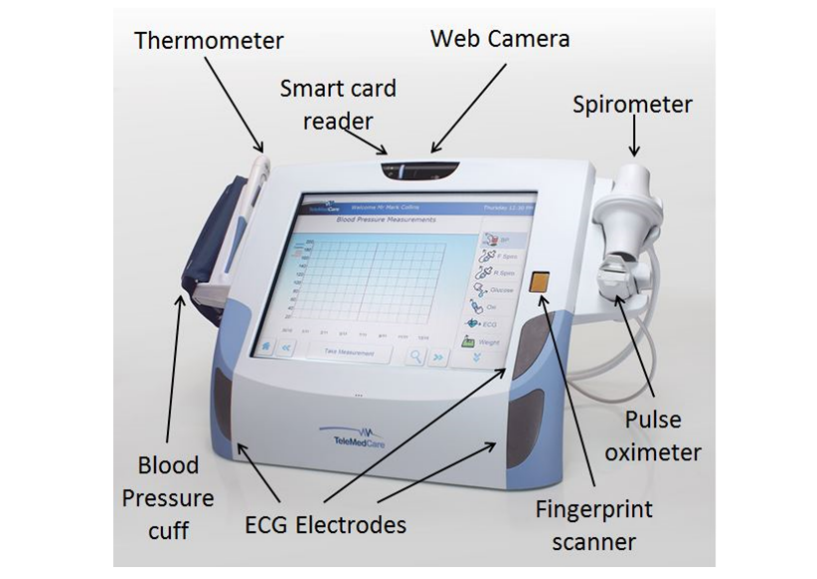
TeleMedCare clinical monitoring unit.

The TeleMedCare (TMC) Systems Clinical Monitoring Unit (TeleMedCare Pty Ltd), depicted in Figure 2, and associated data hosting and clinical web services were selected for the trial; not all features offered by the device were used in this study. The selection of this telemonitoring system was based on the requirements listed below:

- All vital sign measuring devices are part of the system, minimizing issues that can happen by having separate devices connecting to a central unit
- Entire telemonitoring system together with measuring devices and software are approved by the Australian Therapeutic Goods Administration and the US Food and Drug Administration
- Telemonitoring system has no battery requirements as the unit is mains powered
- No incompatibility and calibration issues given the unit is designed to work together with all its devices
- Must be user friendly and easy to operate
- All these factors including subscription costs fit within the time and budget of the trial

The site POs and CCCs configured the telemonitoring system to reflect clinical best practice for the patient’s clinical condition. Patients would be reminded to record some or all of the vital signs shown below, typically in the morning before taking their medications.

- Noninvasive blood pressure (NIBP) using combined oscillometric and auscultatory techniques
- Pulse oximetry to measure arterial blood oxygen saturation
- Single channel ECG, using either the built-in surface electrodes or a custom cable and electrode clamps
- Spirometry, including measurements of vital capacity, peak expiratory flow rate, and volume expired in first second
- Body temperature
- Body weight (SD 100 gm accuracy)
- Glucometer (blood glucose concentration)

In addition to their schedule, patients could take their vital signs at any time. A full suite of clinical questionnaires was also available. These were scheduled and administered by the CCCs and POs.

### Monitoring of Vital Signs

Patients were encouraged to record their vital signs daily. CCCs generally viewed every patient’s record daily, and time spent on each patient was tracked using the CSIRO Web portal and logs implemented on the clinician portal. On average, CCCs accessed the TMC Clinician Web Portal a little more than once a day and spent on average a little less than 30 minutes per week reviewing each patient’s data. There was no significant difference in the time spent by community-based and hospital-based CCCs in the time spent reviewing patient data. CCCs were able to make contact with patients at will to review their progress and discuss the data collected.

Not all patients were required to measure all vital signs. Patients were required to measure vital signs according to their primary disease condition and known comorbidities. Thus 111 patients were monitoring blood pressure, 111 patients were monitoring their peripheral capillary oxygen saturation (SpO_2_), 113 patients were recording their ECG, 52 were recording their blood glucometry, 78 were monitoring their respiratory function, 95 were monitoring their body weight, and 103 were recording their body temperature.

Patients’ adherence with their scheduled daily measurements was calculated by tracking the total number of scheduled events and then counting the actual number of measurement activities completed. Multiple measurements taken on any 1 day are counted as a single measurement. The ratio of these provides a robust measure of adherence.

### Statistical Analysis

Comparisons using the cases available were made between subgroups using the 2-sample *t* test for continuous variables and the Wilcoxon rank-sum test for skewed variables. Baseline characteristics are described using mean and SD for continuous symmetrical variables and mean and 95% CI for skewed data.

Categorical variables are presented as counts and percentages. All statistical tests were 2-tailed, and a *P* value of <.05 was used to indicate statistical significance. Statistical analysis was performed using MATLAB R2016b (MathWorks) and Excel (Microsoft).

### Linear Regression

Linear regression was carried out using the *fit* command in the MATLAB statistics toolbox, and 1-way analysis of covariance (ANCOVA) was used to determine whether the slopes of the linear regression lines are different for the average adherence rates associated with blood pressure, ECG, SpO_2_, blood glucose, body temperature, and body weight for male and female patients.

### Synchronization of Data to Start Date of Telemonitoring

**Figure 3:**
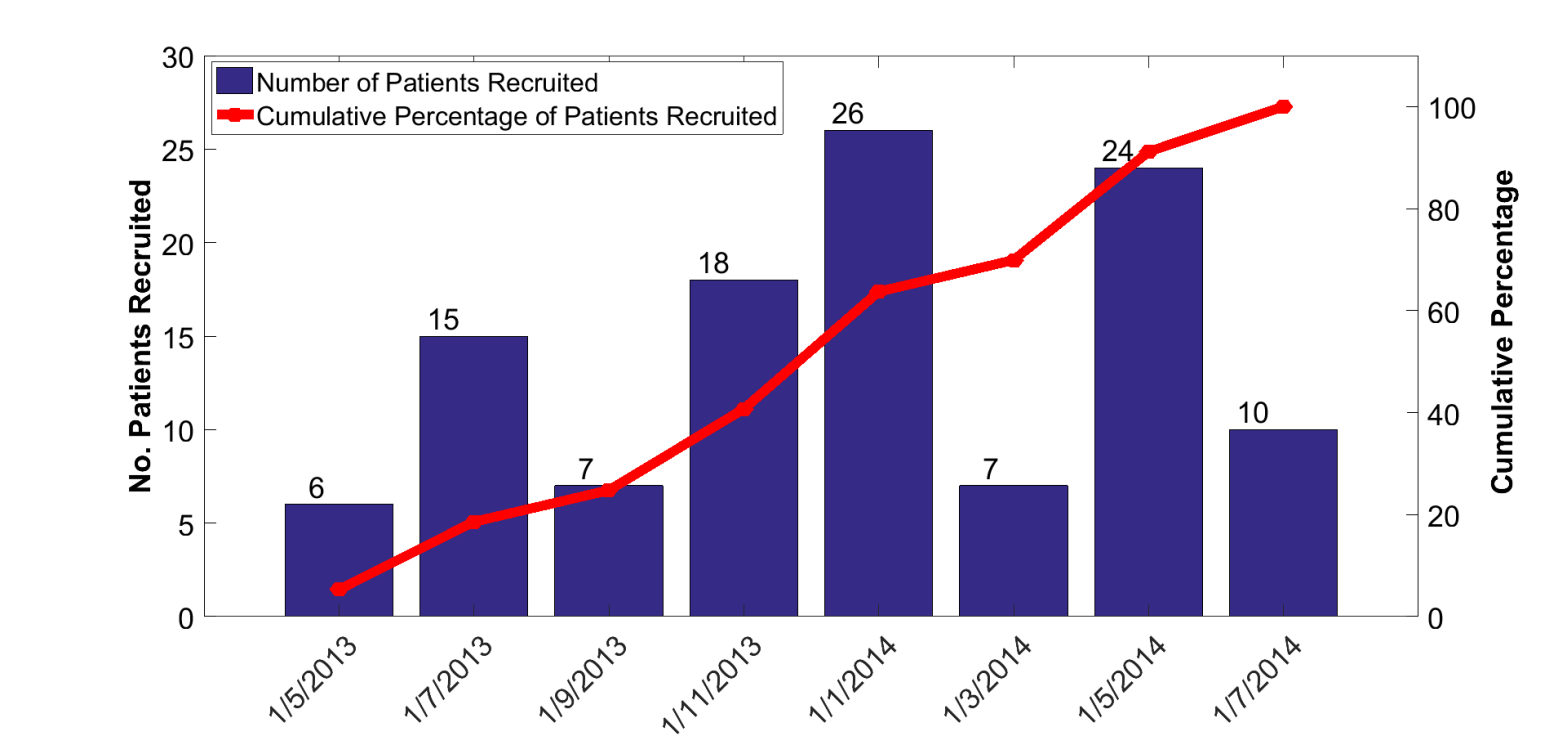
Distribution of commencement dates for monitoring of vital signs.

In this study, time is not calendar time but rather time relative to each patient’s start date of telemonitoring. This compensates for the fact that individual patients were enrolled and commenced telemonitoring over a period of more than a year (Figure 3) and helps to attenuate seasonal effects.

## Results

### Demographics of Test Patients

Basic demographics of test patients in the study are shown in Table 1. There were no significant differences in age between test patients in each of the 5 sites and between male and female patients.

A total of 63.7% (72/113) of the test patients were male and 36.3% (41/113) were female. Most test patients had more than 1 condition listed as a primary diagnosis, and for simplicity, primary disease conditions were grouped in the broad categories of cardiovascular disease (N_Test_=58), respiratory disease (N_Test_=34), and diabetes (N_Test_=21). Figure 3 shows the wide distribution of commencement dates for the test patients arising from local operational delays in identifying patients and providing access to the Internet.

Test patients were monitored on average for 302 days, with no significant difference between average monitoring durations for female patients (287 days) and male patients (310 days); 75% of all test patients were monitored for periods exceeding 6 months. The duration of monitoring for patients who did not drop out was primarily determined by the individual start date of the intervention and the end date of the trial.

### Patient Adherence With Monitoring Schedules

Overall patient adherence data is shown in Table 2. Test patients successfully completed 120,861 measurements of vital signs over a period of 16 months and on average, patients were recording their vital signs with an adherence rate of 64.78%, a little better than once every 2 days.

Patients under the care of community-based CCCs were overall a little less adherent than patients under the care of hospital-based CCCs (63,553/103,737, 61.26%, versus 56,598/81,821, 69.17%). However, adherence with forced spirometry, body temperature, and body weight measurements was substantially greater for patients under hospital-based supervision than for patients supervised in the community. These differences are further explored in the next section.

### Adherence Rates Over Time by Age, Gender, and Mode of Supervision

We now explore differences in adherence rates over time synchronized to the start date of telemonitoring for each patient and between male and female patients and younger and older patients and further analyze the differences observed between hospital-based supervision and community-based supervision. In this analysis, we have only included patients who finished at least 3 months of measurements.

**Table 1:** Basic demographics of test patients at baseline.

Demographics	Hospital-based		Community-based			Total
	TAS^a^	ACT^b^	VIC^c^	NSW^d^	QLD^e^	
Patients, n	29	16	25	17	26	113
Age, years (SD)	68.8 (9.0)	70.1 (8.2)	68.9 (7.6)	76.7 (9.1)	70.0 (10.8)	70.5 (9.3)
Male patients, n	18	11	18	9	16	72
Age, years (SD)	68.9 (10.0)	69.9 (7.7)	69.5 (7.8)	75.4 (7.4)	69.4 (9.0)	70.1 (8.6)
Female patients, n	11	5	7	8	10	41
Age, years (SD)	68.6 (7.4)	70.6 (10.2)	67.4 (7.4)	78.1 (11.2)	70.9 (13.6)	70.1 (8.6)

^a^TAS: Tasmania.

^b^ACT: Australian Capital Territory.

^c^VIC: Victoria.

^d^NSW: New South Wales.

^e^QLD: Queensland.

**Table 2:** Patient adherence with measurement.

Vital signs	Scheduled Items, n	Items completed, n	Adherence, %
Blood pressure	31,117	21,890	70.35
Electrocardiogram	33,719	22,405	63.70
Pulse oximetry	31,102	21,363	68.69
Blood glucose	12,579	8501	67.58
Spirometry	20,498	11,493	56.07
Body temperature	29,792	19,158	64.31
Body weight	27,777	16,051	57.79
Total	186,584	120,861	64.78

We note that in the first quarter of monitoring (see Multimedia Appendix 1), the adherence rates of patients monitored in hospital settings relative to those monitored in community settings were significantly higher for spirometry, blood glucose, body weight, and body temperature. Adherence rates for other measurements and differences in adherence across age and gender were not significantly different.

In the second quarter, significantly higher adherence rates for blood glucose, spirometry, body weight, and body temperature continued to be observed for patients supervised in hospital settings relative to those supervised in community settings, and male adherence with NIBP, SpO_2_, and ECG began to significantly increase relative to female adherence. Adherence with spirometry measurements for the patient cohort older than 70 years also increased significantly (*P*=.049) relative to the 70 years and younger patient cohort.

In the third quarter, males continue to be more adherent than females for NIBP and SpO_2_ measurements, and hospital-supported patients continued to be more adherent than community-supported patients for spirometry measurements.

In the fourth quarter, those aged over 70 years were more adherent than those aged 70 years and younger for spirometry measurements, and males continue to be more adherent than female patients for NIBP, SpO_2_, ECG, and BT. Differences in adherence rates for spirometry between hospital-supported patients (74%, range 64.5% to 83.5%) and community-supported patients (38.6%, 28.6% to 49.1%) become even more pronounced (*P*=.002).

### Linear Regression Analysis of Changes in Adherence Over Time

**Figure 4:**
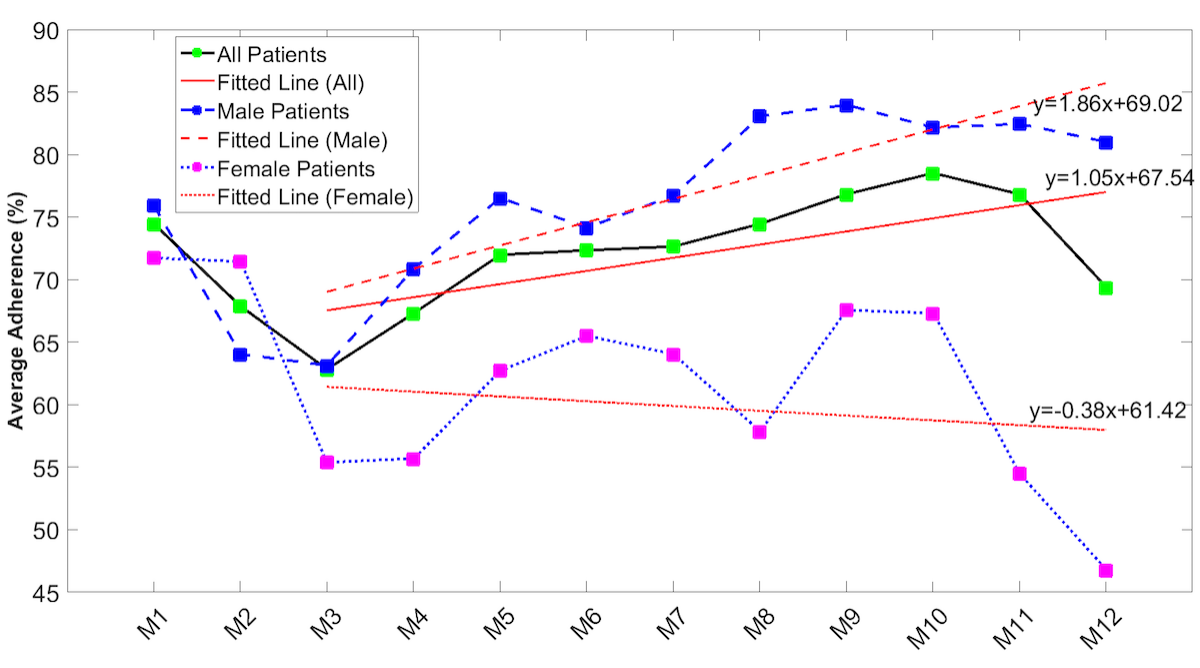
Average adherence rates over time by gender (combined blood pressure, electrocardiogram, peripheral capillary oxygen saturation, blood glucose, body temperature, and body weight). Red lines are linear regression lines (the given equations represent the regression lines).

**Figure 5:**
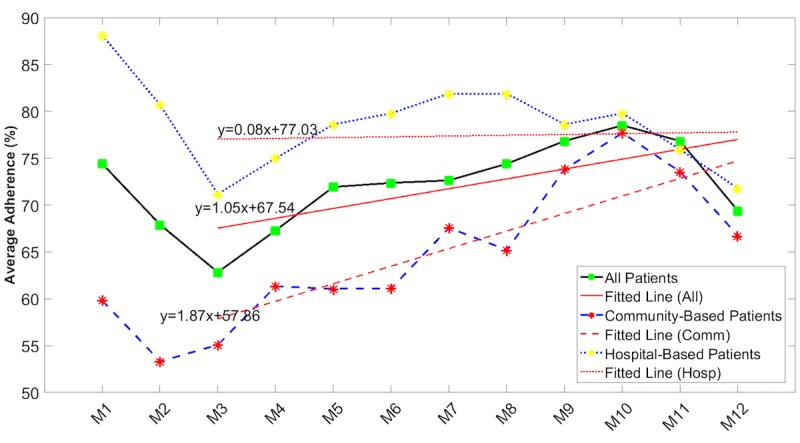
Average adherence rates over time by community-based or hospital-based mode of supervision (blood pressure, electrocardiogram, peripheral capillary oxygen saturation, blood glucose, body temperature, and body weight). Red lines are linear regression lines (the given equations represent the regression equations).

Linear regression was carried out as described in the Methods section with the results shown in Figures 3-5 and Table 3. When adherence rates initially dropped off in the first 2 months of monitoring, these data were removed and analyzed separately to identify long-term trends. ANCOVA analysis comparing slope of the overall male and female adherence data in Figure 4 confirms that there was a significant (*P*=.002) difference in the slopes.

A similar analysis was undertaken for subgroups within the total patient cohort to test whether these results were different for those who were monitored within a community environment or within a hospital environment. In Figure 5, a significant difference (*P*=.02) in slopes for hospital-supervised patients compared with community-supervised patients is observed although the latter appear to increase their adherence over time.

We undertook a similar analysis to test whether these results were different for younger patients (aged 70 years and younger) and older patients (older than 70 years) and for patients with predominantly cardiovascular disease and those with other diseases. Our analysis, however, showed that there were no significant differences between these subgroups.

**Figure 6:**
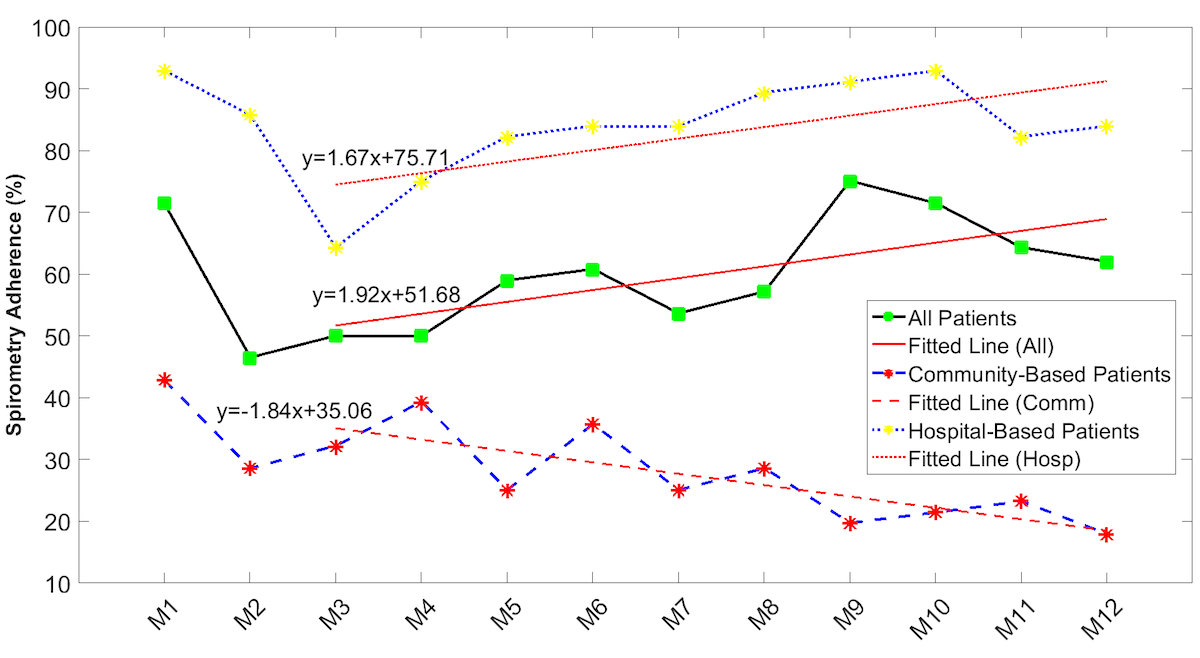
Spirometry adherence rates over time by mode of community-based or hospital-based supervision. Red lines are linear regression lines (the given equations represent the regression lines).

The spirometry adherence rate (Table 4) was an outlier in this analysis in that no significant difference was seen between male and female adherence rate variation over time. However, as shown in Figure 6, while patients supervised in a hospital setting had far higher adherence rates that indeed increased over time, the adherence rates for community-supported patients started at a much lower level and then decreased over time.

**Table 3:** Linear regression and analysis of covariance for different subgroups within the total patient cohort.

Patient subgroups	Slope, %/month (95% CI)	Intercept, % (95% CI)	*P* value
Male patients	1.86 (–0.01 to 3.72)	69.02 (59.05 to 78.79)	.02
Female patients	–0.38 (–2.25 to 1.49)	61.42 (51.45 to 71.40)	—^a^
Hospital-based patients	0.08 (–1.32 to 1.49)	77.03 (69.53 to 84.53)	.02
Community-based patients	1.87 (0.47 to 3.28)	57.86 (50.36 to 65.37)	—
Patients 70 years and younger	0.63 (–0.75 to 2.02)	67.90 (60.51 to 70.30)	.25
Patients older than 70 years	1.40 (0.02 to 2.79)	65.21 (57.82 to 72.61)	—
Cardiac patients	1.53 (–0.16 to 3.22)	68.10 (59.08 to 77.12)	.07
Patients with other chronic conditions	–0.01 (–1.70 to 1.68)	66.04 (57.02 to 75.07)	—

^a^Indicates data is not applicable.

**Table 4:** Linear regression and analysis of covariance for spirometry adherence.

Patient subgroups	Slope, %/month (95% CI)	Intercept, % (95% CI)	*P* value
Male patients	1.81 (0.05 to 3.57)	55.02 (45.64 to 64.41)	.88
Female patients	1.53 (–2.31 to 5.38)	48.40 (27.86 to 68.93)	—^a^
Hospital-based patients	1.67 (0.19 to 3.14)	75.71 (67.85 to 83.58)	<.001
Community-based patients	–1.84 (–3.00 to –0.68)	35.06 (28.87 to 41.26)	—

^a^Indicates data is not applicable.

## Discussion

### Principal Findings

We present in this paper some fundamental results on the ability and willingness of chronically ill patients to engage with a comprehensive regime of at-home telemonitoring of their vital signs over extended periods of time, in some cases exceeding a year. In our study there were no significant differences in health literacy, socioeconomic status, or level of previous experience with technology. We recognize that different vital signs are easier and more acceptable than others to carry out, and we anticipated that overall adherence rates would reflect the ease of use and perceived value of the measurement. Overall adherence rates were, however, surprisingly similar, ranging from 56.1% for spirometry to 70.4% for blood pressure measurements. This is consistent with the relative complexity of spirometry measurements, which require 3 forced expiratory efforts and could be quite tiring for frail, chronically ill patients.

Our results straddle levels of adherence reported by Maeder [6] of between 40% to 90% and do show an initial steady drop-off over time, particularly in the first few weeks or months after the start. Unlike Port et al [29], who reported that an initial adherence rate of 89% dropped to around 50% within 10 months, our study shows an increase in adherence following an initial drop. Our study also confirms that higher adherence rates are reported for simple types of monitoring and for shorter periods of intervention [6].

Generally, adherence with measurement of vital signs fell over the first 3 months (Multimedia Appendix 1 and Figures 4 and 5) but recovered over time to levels achieved at the start of monitoring. As a result, there were no significant differences between adherence rates recorded in the first quarter of monitoring and those recorded in the last quarter of monitoring. Measurement of spirometry by women, however, started from a very low level and continued to fall over time (Figure 6). Age did not appear to be a factor, as there were no significant differences between adherence rates of those aged 70 years and younger and those older than 70 years. Gender, however, was a factor, as male subjects were more adherent than female patients across all measurement modalities. The differences were only significant for the measurement of NIBP, SpO_2_, and ECG (Multimedia Appendix 1). These data on gender differences are in good agreement with the literature, which reports reduced adherence by women to telemonitoring of BP [30], antiretroviral therapies [31], management of cystic fibrosis [32], and medications [33].

In 2 of our sites, care coordination was hospital-based and was carried out by highly trained specialist nurses. In the other 3 sites, care coordination was carried out by community nurses often without any additional clinical support. Patients supported by hospital-based care coordinators were significantly more adherent with their measurement of lung function (spirometry), blood glucose, body weight, and body temperature than those supported by community-based care coordinators. Adherence was also higher for patients supported by hospital-based care coordination for NIBP, SpO_2_, and ECG, but the differences were not significant.

We cannot fully explain the initial fall of adherence in the first quarter other than to hypothesize that CCCs may be more successful in encouraging patients to continue with their measurements once a longitudinal patient record is achieved and its value noted. The other major conclusion of this study is that care coordination by hospital-based staff leads to higher levels of adherence than for patients managed by community-based staff. This would suggest that hospital-based staff may be more cognizant of the benefits of monitoring vital signs, more capable of interpreting the longitudinal patient record, and therefore more willing to offer greater encouragement and support to their patients.

According to the systematic review undertaken by Maeder et al [6], the adherence rate usually dropped off steadily over time in most of the studies, and this drop is more evident in the first few weeks or months after the start. Although it is evident from our results that the overall adherence rate dropped off in the first 3 month, it did not continue to fall and indeed recovered over time. We can therefore conclude that chronically ill patients are able to monitor their vital signs at least once every 2 days over prolonged periods of time even for the most difficult measurements such as spirometry and ECG.

Our results also found that the adherence rates in male and community-based subgroups of patients significantly increased over time while the adherence rates in female and hospital-based patients slightly decreased over time. Additionally, the adherence rate variations over time were not significantly different between younger and older subgroups within the total patient cohort or between patients with predominantly cardiovascular disease and those with other diseases.

The telemonitoring system used in this study had some unique features that may have encouraged increased patient adherence with the vital sign measurements. During the recording of a vital sign, the underlying graphical data such as the pressure trace, oscillometric waveforms, and Korotkoff sounds for NIPB measurements were visible to patients and may have promoted greater adherence and recording of better quality traces. Patients were also able to view their own longitudinal records on screen and could respond to increasing and decreasing trends in their data. More research, however, needs to be carried out on the relative importance of user interfaces and the quality of biomedical instrumentation in maintaining the interest and involvement both of patients and the CCCs.

### Limitations

Limitations of this study include the reduced numbers of patients who were monitored for the full 12 months, dropping, for example, from 105 patients recording NIBP in the first quarter to 61 in the fourth quarter (as reported in Multimedia Appendix 1). This drop, however, is the result of the fact that patients began their monitoring over a period of more than 6 months and all monitoring ended when the trial ended. As a result, some test patients were monitored for just a little more than 6 months and others for more than 12 months. In addition, the ability of CCCs to support and encourage patients to continue with their telemonitoring of vital signs was not rigorously evaluated and was probably quite variable, although we did confirm that generally patients supported by hospital-based specialist nurses had higher adherence with their measurement schedules.

In a previous paper, we reported that test subjects could be characterized as having the following primary chronic conditions: 50% cardiac disease (mainly CHF), 30% respiratory disease (mainly COPD), and the remaining 20% diabetes [8]. All subjects had some comorbidities. As stated earlier, in choosing a population health approach, we analyze the total patient cohort as a homogeneous group. We recognize, however, that some additional insights could result from analyzing and comparing adherence based on the primary diagnosis.

### Conclusions

We have previously reported the following outcomes for this trial: significant drop in expenditure on medical services, reduced admissions to the hospital, and significant reduction in hospital length of stay [8]. Data presented here suggest that levels of adherence to long-term telemonitoring by patients with chronic conditions support the hypothesis that longitudinal recording of vital signs data provides powerful tools for early identification of an exacerbation of a patient’s condition and may permit the early mobilization of clinical resources to avoid unnecessary hospitalization [8].

## Appendix

Multimedia Appendix 1 Adherence rates over time as a function of gender, age, and supervisory setting.

## Abbreviations

ACT: Australian Capital Territory
ANCOVA: analysis of covariance
CCC: clinical care coordinator
CHF: congestive heart failure
COPD: chronic obstructive pulmonary disease
CSIRO: Commonwealth Scientific and Industrial Research Organisation
ECG: electrocardiogram
NIBP: noninvasive blood pressure
NSW: New South Wales
QLD: Queensland
SpO _2_: peripheral capillary oxygen saturation
TAS: Tasmania
TMC: TeleMedCare
VIC: Victoria
