# Supplementary material for: Patient Adherence to Scheduled Vital Sign Measurements During Home Telemonitoring: Analysis of the Intervention Arm in a Before and After Trial
Source: JMIR Med Inform. 2018 Apr 9;6(2):e15. doi: 10.2196/medinform.9200 (PMC5913569; doi:10.2196/medinform.9200)
Supplement: Multimedia Appendix 1 [file medinform_v6i2e15_app1.pdf]

Adherence rates over time as a function of gender, age, and supervisory setting.

Significant differences are indicated by <.05*, <.01** and <.001***

	ALL	M	F	*P*	Age<br>≤ 70	Age<br>> 70	*P*	Hosp<br>Based	Comm<br>Based	*P*
Panel A 0-3 months										
NIBP<br>(%)	67.7<br>(62.6-72.9)<br>N=105	69.4<br>(63.0-75.8)<br>N=68	64.7<br>(55.9-73.6)<br>N=37	.35	70.5<br>(62.8-78.2)<br>N=49	65.4<br>(58.4-72.4)<br>N=56	.25	72.5<br>(64.0-81.1)<br>N=39	64.9<br>(58.4-71.4)<br>N=66	.11
SpO_2<br>(%)	65.6<br>(60.4-70.9)<br>N=104	67.8<br>(61.6-74.0)<br>N=67	61.7<br>(52.3-71.2)<br>N=37	.35	71.0<br>(63.7-78.3)<br>N=48	61.0<br>(53.8-68.3)<br>N=56	.054	71.9<br>(64.0-79.7)<br>N=38	62.1<br>(55.3-68.8)<br>N=66	.09
ECG<br>(%)	64.7<br>(59.4-70.0)<br>N=107	67.3<br>(61.2-73.4)<br>N=69	60.0<br>(50.0-70.0)<br>N=38	.31	67.8<br>(59.9-75.7)<br>N=50	62.0<br>(54.9-69.2)<br>N=57	.21	70.2<br>(61.6-78.8)<br>N=41	61.3<br>(54.7-67.9)<br>N=66	.06
BGL<br>(%)	53.9<br>(48.0-59.8)<br>N=46	55.9<br>(49.7-55.9)<br>N=33	48.9<br>(35.2-62.5)<br>N=13	.62	58.8<br>(51.3-66.3)<br>N=24	48.7<br>(39.8-57.5)<br>N=22	.10	65.1<br>(58.0-72.3)<br>N=11	50.4<br>(43.3-57.5)<br>N=35	.03*
Spiro<br>(%)	53.0<br>(45.4-60.6)<br>N=67	52.9<br>(43.9-61.9)<br>N=42	53.1<br>(39.4-66.9)<br>N=25	.96	50.0<br>(38.7-61.4)<br>N=32	55.7<br>(45.5-66.0)<br>N=35	.48	69.5<br>(59.4-79.5)<br>N=27	41.9<br>(32.6-51.2)<br>N=40	<.001***
BW<br>(%)	54.4<br>(47.2-61.5)<br>N=87	51.8<br>(42.9-60.7)<br>N=61	60.5<br>(48.8-72.1)<br>N=26	.46	54.8<br>(43.5-66.0)<br>N=41	54.0<br>(44.8-63.3)<br>N=46	.72	67.1<br>(57.9-76.4)<br>N=39	44.0<br>(34.3-53.7)<br>N=48	.004**
BT<br>(%)	63.0<br>(57.0-68.9)<br>N=91	64.4<br>(57.3-71.6)<br>N=59	60.2<br>(49.5-70.9)<br>N=32	.48	68.3<br>(60.4-76.2)<br>N=47	57.2<br>(48.4-66.0)<br>N=44	.07	70.8<br>(63.0-78.6)<br>N=40	56.8<br>(48.4-65.2)<br>N=51	.04*
Panel B 3-6 months										
NIBP<br>(%)	65.6<br>(58.9-69.9)<br>N=105	70.4<br>(63.8-77.1)<br>N=68	56.9<br>(47.0-66.8)<br>N=37	.01*	63.7<br>(54.9-72.5)<br>N=49	74.7<br>(60.0-67.3)<br>N=56	.79	71.1<br>(61.9-80.2)<br>N=39	62.4<br>(55.3-69.6)<br>N=66	.08
SpO_2<br>(%)	64.9<br>(71.7-82.6)<br>N=104	69.2<br>(62.7-75.7)<br>N=67	55.7<br>(46.0-65.5)<br>N=37	.02*	64.9<br>(56.3-73.4)<br>N=48	64.0<br>(56.7-71.4)<br>N=56	.71	71.3<br>(62.9-79.7)<br>N=38	60.4<br>(53.3-67.6)<br>N=66	.06
ECG<br>(%)	64.1<br>(58.5-69.6)<br>N=107	68.4<br>(61.9-75.0)<br>N=69	49.1<br>(34.3-63.9)<br>N=25	.03*	63.9<br>(55.8-63.9)<br>N=50	64.2<br>(56.5-71.9)<br>N=57	.95	70.5<br>(62.0-79.1)<br>N=41	60.1<br>(52.9-67.2)<br>N=66	.06
BGL<br>(%)	54.5<br>(47.6-61.4)<br>N=46	57.1<br>(49.6-64.6)<br>N=33	47.9<br>(32-7-63.0)<br>N=13	.29	58.1<br>(48.6-67.6)<br>N=24	50.6<br>(40.6-60.5)<br>N=22	.16	69.7<br>(62.8-75.5)<br>N=11	49.7<br>(41.5-57.9)<br>N=35	.02*
Spiro<br>(%)	52.4<br>(44.0-60.9)<br>N=67	54.4<br>(44.1-64.7)<br>N=42	49.1<br>(34.3-63.9)<br>N=25	.40	42.8<br>(30.8-54.8)<br>N=32	61.2<br>(49.9-72.5)<br>N=35	.049*	70.4<br>(58.9-81.8)<br>N=27	40.3<br>(29.9-50.7)<br>N=40	<.001***
BW<br>(%)	54.7<br>(47.2-62.1)<br>N=87	54.9<br>(45.8-63.9)<br>N=61	54.1<br>(40.5-67.8)<br>N=26	.57	49.2<br>(37.9-60.6)<br>N=41	59.5<br>(49.7-62.3)<br>N=46	.20	65.0<br>(54.7-75.3)<br>N=39	46.2<br>(36.1-56.4)<br>N=48	.03*
BT<br>(%)	61.0<br>(54.7-67.3)<br>N=91	62.6<br>(54.7-70.5)<br>N=59	58.0<br>(47.7-68.4)<br>N=32	.23	61.6<br>(52.6-70.7)<br>N=47	60.3<br>(51.6-69.0)<br>N=44	.60	69.7<br>(61.8-77.5)<br>N=40	54.2<br>(45.2-63.1)<br>N=51	.04*

Panel C 6-9 months										
NIBP (%)	67.9 (62.3-73.5) N=83	74.6 (68.1-74.6) N=53	56.1 (46.6-65.7) N=30	.006**	64.7 (56.2-73.3) N=38	70.6 (63.2-78.0) N=45	.44	72.2 (63.2-81.2) N=35	64.8 (57.6-72.0) N=41	.20
SpO_2 (%)	66.7 (61.1-72.4) N=82	73.1 (66.5-79.7) N=53	69.7 (60.5-78.8) N=29	.007**	64.2 (55.5-72.9) N=38	68.9 (61.6-76.3) N=44	.61	69.3 (59.8-78.8) N=36	64.7 (57.7-71.7) N=46	.39
ECG (%)	65.7 (60.1-71.2) N=84	69.3 (62.2-76.5) N=54	59.1 (50.4-67.7) N=30	.07	62.7 (54.2-71.3) N=39	68.2 (60.9-75.5) N=45	.45	71.5 (63.2-79.8) N=37	61.1 (53.7-68.4) N=47	.10
BGL (%)	56.9 (51.0-62.7) N=30	59.5 (52.7-66.4) N=22	49.7 (38.6-60.8) N=8	.29	56.8 (48.1-65.5) N=19	57.1 (49.4-64.7) N=11	.90	65.5 (57.4-73.6) N=10	52.6 (45.4-59.9) N=20	.23
Spiro (%)	52.8 (44.1-61.4) N=55	56.2 (45.2-67.2) N=36	46.2 (32.3-46.2) N=19	.35	44.7 (32.8-56.7) N=25	59.4 (47.4-71.5) N=30	.24	69.0 (56.3-81.7) N=26	38.2 (28.3-48.1) N=29	<.001***
BW (%)	59.9 (52.6-67.3) N=71	63.8 (55.0-72.6) N=48	51.7 (38.7-64.8) N=23	.14	55.8 (44.7-66.9) N=30	62.9 (53.0-72.8) N=41	.46	67.1 (57.1-77.0) N=35	52.9 (42.5-63.4) N=36	.15
BT (%)	66.3 (60.2-72.4) N=75	68.9 (60.7-77.1) N=48	61.7 (53.1-70.2) N=27	.19	65.1 (56.7-73.5) N=38	67.6 (58.7-76.6) N=37	.53	71.5 (62.9-80.0) N=37	61.3 (52.8-69.8) N=38	.12
Panel D 9-12 months										
NIBP (%)	68.2 (62.9-73.5) N=61	74.4 (69.1-79.7) N=41	55.4 (44.7-66.2) N=20	.04*	63.1 (55.5-70.7) N=31	73.4 (66.2-80.7) N=30	.07	69.6 (60.6-78.6) N=28	67.0 (60.4-73.5) N=33	.71
SpO_2 (%)	67.9 (62.8-73.0) N=61	74.6 (69.8-79.5) N=41	54.2 (43.6-64.8) N=20	.03*	62.6 (55.0-70.3) N=31	73.4 (66.7-80.1) N=30	.08	70.0 (61.6-78.3) N=28	66.2 (59.6-72.8) N=33	.65
ECG (%)	64.9 (59.6-70.2) N=66	70.5 (64.1-76.8) N=43	54.5 (45.5-63.4) N=23	.02*	61.8 (54.0-69.7) N=32	67.8 (60.5-75.1) N=34	.42	63.9 (54.7-73.1) N=33	65.9 (59.5-72.3) N=33	.92
BGL (%)	56.8 (50.2-63.4) N=20	60.9 (53.1-68.6) N=14	47.3 (35.3-59.3) N=6	.15	59.3 (50.0-68.7) N=12	53.0 (43.4-62.6) N=8	.30	59.1 (47.0-71.1) N=8	55.3 (47.0-63.6) N=12	.97
Spiro (%)	56.8 (48.5-65.0) N=41	57.1 (46.6-67.5) N=29	56.0 (42.2-69.8) N=12	.84	47.7 (36.4-58.9) N=21	66.3 (54.9-77.7) N=20	0.047*	74.0 (64.5-83.5) N=21	38.6 (28.6-49.1) N=20	0.002**
BW (%)	56.7 (49.2-64.2) N=56	61.5 (52.7-70.2) N=39	45.7 (31.8-59.6) N=17	.054	53.4 (41.6-65.2) N=24	59.1 (49.3-68.9) N=32	.59	63.9 (53.4-74.4) N=30	48.3 (37.8-58.8) N=26	.11
BT (%)	62.3 (56.1-68.5) N=61	67.8 (60.0-75.5) N=39	52.6 (42.9-62.3) N=22	.03*	60.1 (51.8-68.3) N=31	64.6 (55.2-74.0) N=30	.44	64.3 (54.9-73.8) N=33	59.9 (51.6-68.2) N=28	.67
